# Comparison of Microgravity Analogs to Spaceflight in Studies of Plant Growth and Development

**DOI:** 10.3389/fpls.2019.01577

**Published:** 2019-12-06

**Authors:** John Z. Kiss, Chris Wolverton, Sarah E. Wyatt, Karl H. Hasenstein, Jack J.W.A. van Loon

**Affiliations:** ^1^Department of Biology, University of North Carolina—Greensboro, Greensboro, NC, United States; ^2^Department of Botany & Microbiology, Ohio Wesleyan University, Delaware, OH, United States; ^3^Molecular and Cellular Biology Program, Department of Environmental & Plant Biology, Ohio University, Athens, OH, United States; ^4^Biology Department, University of Louisiana at Lafayette, Lafayette, LA, United States; ^5^DESC (Dutch Experiment Support Center), Department of Oral and Maxillofacial Surgery/Oral Pathology, Amsterdam University Medical Center, Amsterdam, Netherlands; ^6^Academic Centre for Dentistry Amsterdam (ACTA), VU-University, Amsterdam, Netherlands; ^7^European Space Agency (ESA) Technology Center (ESTEC), Life & Physical Science, Instrumentation and Life Support Laboratory, TEC-MMG, Noordwijk, Netherlands

**Keywords:** *Arabidopsis*, clinostat, plant growth, simulated microgravity, random positioning machine, reduced gravity, spaceflight experiments

## Abstract

Life on Earth has evolved under the influence of gravity. This force has played an important role in shaping development and morphology from the molecular level to the whole organism. Although aquatic life experiences reduced gravity effects, land plants have evolved under a 1-*g* environment. Understanding gravitational effects requires changing the magnitude of this force. One method of eliminating gravity'’s influence is to enter into a free-fall orbit around the planet, thereby achieving a balance between centripetal force of gravity and the centrifugal force of the moving object. This balance is often mistakenly referred to as microgravity, but is best described as weightlessness. In addition to actually compensating gravity, instruments such as clinostats, random-positioning machines (RPM), and magnetic levitation devices have been used to eliminate effects of constant gravity on plant growth and development. However, these platforms do not reduce gravity but constantly change its direction. Despite these fundamental differences, there are few studies that have investigated the comparability between these platforms and weightlessness. Here, we provide a review of the strengths and weaknesses of these analogs for the study of plant growth and development compared to spaceflight experiments. We also consider reduced or partial gravity effects *via* spaceflight and analog methods. While these analogs are useful, the fidelity of the results relative to spaceflight depends on biological parameters and environmental conditions that cannot be simulated in ground-based studies.

## Introduction

Plants have evolved under the influence of Earth'’s gravity, a force of “1 *g*.” This ubiquitous force affects plant growth, development, and morphology at all levels, from the molecular to the whole plant (Vandenbrink et al., 2014). In addition, gravity underlies other physical phenomena like buoyancy, convection, and sedimentation, which affect many physical and chemical processes and therefore also shape plant growth and development. For example, buoyancy affects gas exchange, cellular respiration, and photosynthesis, but itself is a function of varying densities (Braun et al., 2018).

Studying the direct and indirect effects of gravity on plant growth, however, is complicated by the difficulty of changing gravity on Earth. One means of reducing gravity'’s influence is to establish free fall and eliminate the effect of gravity either for a few seconds in so-called drop towers and parabolic flights or for the long term by using orbital free fall, which creates weightlessness. This condition is achieved by the balance between Earth'’s gravity and the velocity required to maintain free fall (Kiss, 2015). Experiments focusing on plant growth and development have been carried out in this environment almost from the advent of human spaceflight in the 1960s (Wolverton and Kiss, 2009; Vandenbrink and Kiss, 2016).

Fascinating insights into plant biology have been provided by spaceflight studies aboard orbiting spacecraft. For instance, at the cell/molecular level, changes in the cell cycle (Manzano et al., 2009; Matía et al., 2010) and the cell wall (Soga et al., 2002; Johnson et al., 2015) have been observed when plants develop in microgravity. Recently, there have been a plethora of spaceflight experiments on the effects of varying gravity levels on gene expression in plants (Paul et al., 2012; Correll et al., 2013; Kwon et al., 2015; Johnson et al., 2017; Paul et al., 2017; Choi et al., 2019). And facilitated by the absence of significant gravitational accelerations in spaceflight, novel mechanisms of phototropism (Molas and Kiss, 2009) have been discovered in flowering plants (Millar et al., 2010; Kiss et al., 2012; Vandenbrink et al., 2016). On the applied side of plant space research, there has also been progress on cultivating plants for use in bioregenerative life support systems (Braun et al., 2018).

Because of the scarcity of access to spaceflight, researchers have used other approaches to minimize or eliminate constant 1-*g* conditions (Kiss, 2015). These methods include drop towers (samples are weightlessness for seconds), parabolic flights in specialized airplanes (samples are weightlessness for approximately 10–20 s), and sounding rockets (minutes of weightlessness) as attractive alternatives (see also Beysens and van Loon, 2015). Sounding rockets are retrieved in the same general area after their launch without entering into orbit. In the free-fall phase, these missions typically provide 3–8 min of microgravity (Böhmer and Schleiff, 2019). In recent years, private companies such as Blue Origin and Virgin Galactic are promising suborbital flight with several minutes of microgravity (Pelton, 2019). However, for most systems in plant biology, these suborbital methods provide a period of weightlessness that is too short to effectively assay growth and development. A conceptual alternative to these methods of creating brief free-fall conditions is to develop conditions in which the direction of the gravity vector is constantly changing through the use of clinostats and similar devices.

## Clinostats

Clinostats have been developed since gravity was identified as a major contributor of plant growth and development by Knight, Sachs, and Ciesielski in the late 1800s (reviewed in Hoson et al., 1997; van Loon 2007; Hasenstein, 2009; Herranz et al., 2013). A clinostat is a device that rotates specimens around one or more axes. A number of different types of clinostats have been used to study plant growth and development as well as to address basic issues in fundamental biology. These clinostats can be divided into several types: one-axis clinostats with slow (1–4 rpm) or fast (50–120 rpm) rotation and clinostats with two or three axes of rotation. If the rate of rotation for the axes varies, such systems are distinguished as random positioning machines (RPMs). In addition to these instruments, magnetic levitation has been used to balance gravity (Kamal et al., 2016).

### One-Axial Clinostats

The first experiments to expose plants to altered gravity environments were performed nearly 160 years before humans reached low-Earth orbit. Early in the 19th century, T.A. Knight used a water wheel as a centrifuge to expose oat seedlings to variable acceleration, demonstrating that plants were indeed sensing this physical force when carrying out “geotropic” growth (Knight, 1806). Later in the same century, Sachs developed a device, which he named a “klinostat,” to alter the *effects* of gravity by constantly rotating its longitudinal axis horizontally, thereby averaging the presumed effect of the gravitational force over the rotated axis (Sachs, 1882).

**Figure 1:**
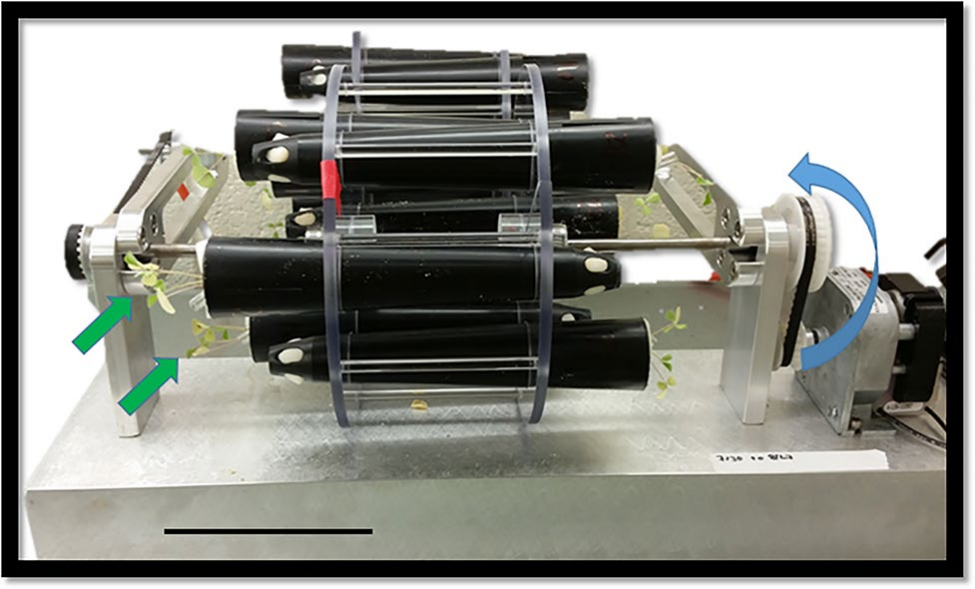
A standard two-dimensional clinostat used to grow *Medicago* seedlings (*green arrows*) at 1 rpm. *Blue arrow* indicated the direction of rotation. *Scale bar*, 10.5 cm.

Clinostats have been employed as a control for the gravitational force in numerous studies investigating plant development and responses to directional stimuli (**Figure 1**). The clinostat has frequently been used for studies in which the researcher wished to reorient the organ or cell in the gravitational field for a period of time, then eliminate, as much as possible, the influence of constant gravity on the organ. Such was the use of clinostats in experiments investigating both the presentation time, perception time, and lag time of the gravitropic response of various species and organs (Pickard 1973; Johnsson and Pickard, 1979; Kiss et al., 1989). The theoretical justification for the use of clinostats was elaborated by Dedolph and Dipert (1971); they demonstrated the importance of the rotation rate on the effectiveness of the clinostat due to its influence on the sedimentation path of the starch statoliths, thought to be the primary means of gravity susception in plants (Kiss, 2000). They found that rotation rates of 2–4 rpm corresponded to a more effective randomization because it minimized the path length of statolith sedimentation.

In addition to their use as a means of minimizing the unidirectional effects of gravity, several studies have incorporated modified versions of the clinostat that expose the axial organ to a fractional *g* treatment either through a programmed rotation pattern or through the incorporation of a centrifuge as the innermost rotating axis of the clinostat. These instruments have been key in estimating the threshold acceleration necessary to activate gravity perception and growth responses (Shen-Miller et al., 1968; Brown et al., 1995; Laurinavicius et al., 1998; Galland et al., 2004; Duemmer et al., 2015; Bouchern-Dubuisson et al., 2016; Frolov et al., 2018), as well as identifying cellular-level responses of plants to microgravity (Murakami and Yamada, 1988; Kraft et al., 2000; Dauzart et al., 2016; Manzano et al., 2018) and the persistence of the gravity stimulus (John and Hasenstein, 2011).

Despite their usefulness for temporarily changing the unidirectional force of gravity, there is also evidence that such treatments introduce their own sets of stimuli that may compete with or confound interpretation of those pathways of most interest to the user (Hasenstein and van Loon, 2015). Centrifugal forces resulting from rotation about one or two axes varies as a function of the position of the organ under study along the radius and speed of rotation, and the organ will experience variable *g* levels across its axis. Because the force of gravity itself is never altered, the bending due to differential growth will cause the position of the organ with respect to the radius of rotation to change over the course of an experiment. For example, the growth of an axial organ over the course of a long-term experiment will result in the organ experiencing a change in acceleration if the growth direction is away from the center of the axis of rotation. This factor is one source of complexity when interpreting the results of clinostat experiments, as indicated by the observation that plants respond differently when rotated around one axis versus the other (John and Hasenstein, 2011; Hasenstein and van Loon, 2015).

Long-term experiments on clinostats are particularly challenging because as the organs increase in mass, the changing weight distribution will cause bending stresses and other non-random mechanical stimulation that will vary as a function of the specific load-bearing structure of each organ. Thus, growing plants on a rotating clinostat can result in mechanical stress (van Loon, 2007; Manzano et al., 2009). The use of clinostats to study developmental effects of gravity are also limited because of their inability to control for constantly changing loads and rotational forces, thus restricting their usefulness with plants mainly to studies of directional growth responses. Thus, many factors, such as weighting distribution and rotation velocity, need to be considered when designing clinostats for life science experiment (Brown et al., 1996).

Despite these disadvantages, the simplicity and availability of clinostats are the main reasons that these devices are the most common approach to attempt to simulate altered gravity conditions. Although the artifacts associated with clinostats require caution of the assessment of gravitational effects, they can provide valuable comparisons with space experiments and have been widely used by many researchers (e.g., Brown et al., 1995; Kraft et al., 2000).

### Fast-Rotating Clinostats

Slow-rotating clinostats as described above simply consider the overall geometry and develop a scheme of rotation that fulfills certain conditions (such as centrifugal accelerations less than 10^−3^ *g*). However, fast-rotating clinostats (typically 50–120 rpm) also utilize the path of sedimentation in a fluid, usually an aqueous growth medium for small (<1 mm) organisms (Aleshcheva et al., 2016; Warnke et al., 2016).

In liquids, sedimentation and a relatively slow rotation result in potentially significant artifacts including spirally movements from centrifugation, sedimentation, and a viscosity-dependent Coriolis force. When the speed of rotation is increased as in a fast-rotating clinostat, sedimentation of a particle will be less than the movement of the liquid, thereby resulting in a reduced radius that finally produces a smaller diameter than the size of the particle or a cell. Thus, in the conditions as found in the fast-rotating clinostat, the rotation stabilizes the fluid around the particle, which in turn eliminates the gravity effects for all practical purposes. While the fast-rotating clinostat can provide conditions that mimic weightlessness very well, it is limited to small organisms such as unicells or bacteria but, generally, not applicable for plant studies (Cogoli, 1992).

### Non-Uniformly Rotating Clinostats

**Figure 2:**
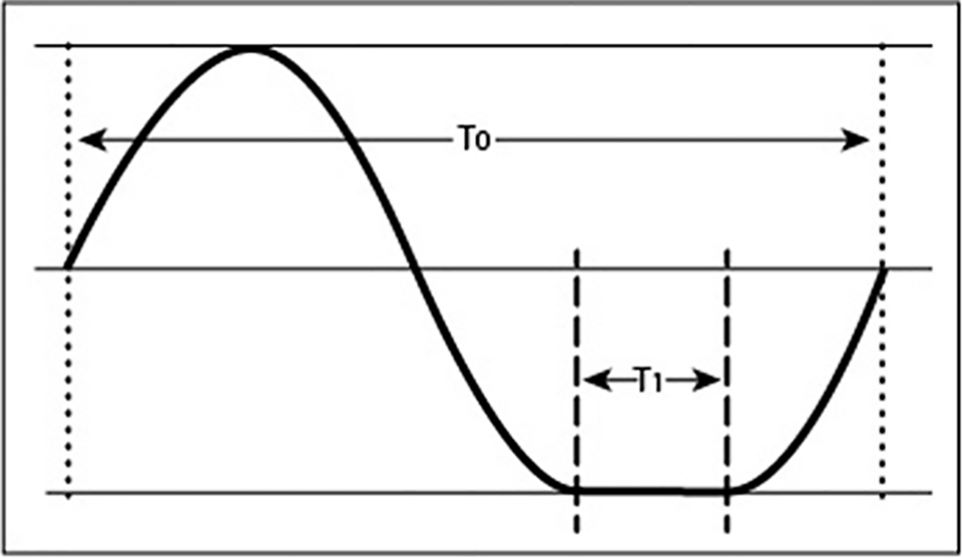
Motion profile of a an object experiencing fractional gravity as a result of non-uniform rotation. The trace of a point rotating around an axis shows an extended resting position during phase T_1_. The ratio between the dwell time in the bottom position (T_1_) and complete rotation (T_0_) corresponds to the fractional gravity experienced by plants, provided that the dwell time does not exceed the gravity perception time.

In addition to positioning one-axial clinostats at certain angles to mimic fractional gravity levels (<1 *g*), it is possible to achieve a similar condition by changing the rate of horizontal rotation such that the rotation is stopped during the bottom time (Brungs et al., 2016). The bottom dwell time determines the effective residual acceleration. When uniform rotation represents complete gravity compensation for a 1-rpm (∼0.1 rad s^−1^) clinostat, extending each rotation by the amount of gravity that is supposed to be established, for example 0.1 g, would require a bottom dwell time of 6 s. The extra 6 s relative to the normal rotation of 60 s (=1 rpm) spent at the “bottom” position (**Figure 2**) creates 0.1 *g* net acceleration. Because additional acceleration or deceleration needs to be minimized, the movement requires precise algorithms and motor control. The advantage of such designs is that fractional *g*-levels can be established. Nonetheless, this principle also depends on rotation and therefore suffers from the same shortcomings as standard clinostats (Hasenstein and van Loon, 2015).

### Random Positioning Machines

**Figure 3:**
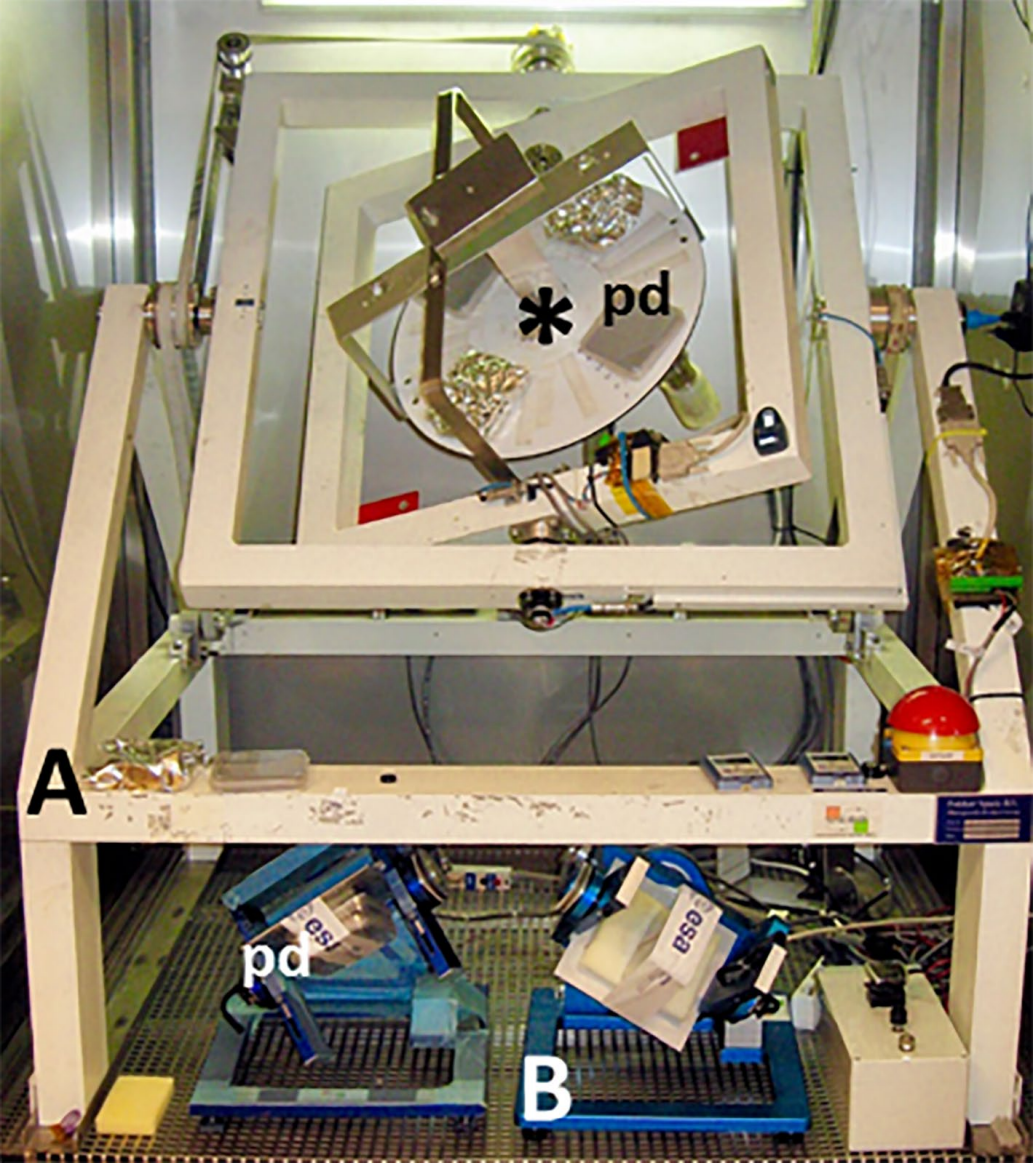
Three random positioning machines (RPMs) each with two independently driven perpendicular frames. The discrete rotation axes allow the implementation of slip rings to provide power and exchange data with the experiment that can be mounted onto the inner frame. Both the full-sized RPM **(A)** and the two desktop models **(B)** are shown with 10-cm square Petri dishes (*pd*). The diameter of the disk (*asterisk*) on the full-sized RPM is 40 cm and provides the generation of partial gravity.

**Figure 4:**
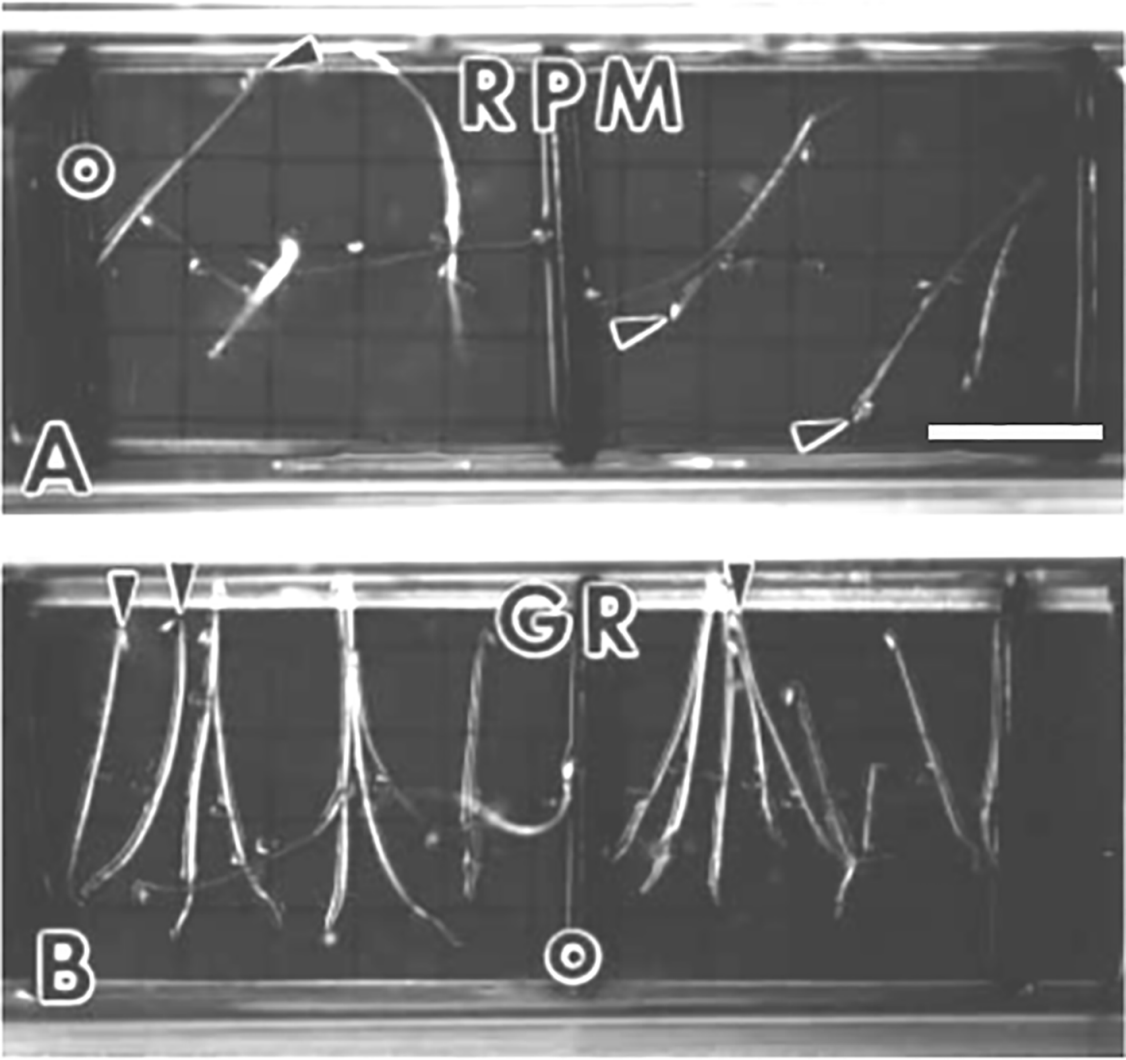
*Arabidopsis* seedlings grown in spaceflight hardware for 3.5 days in the dark. *Arrowheads* indicate the hypocotyl apex. **(A)** Seedlings that germinated and developed on the random positioning machine (*RPM*) are disoriented. **(B)** Ground controls (*GR*) are oriented to the gravity vector which is toward the bottom of the photograph. *Scale bar*, 6 mm. Figure is from Kraft et al. (2000) and is used with permission from Springer Nature publishers.

The limited ability to average gravity effects by horizontal rotation led to the evolution of RPMs in order not to generate constant accelerations in any particular direction (Kraft et al., 2000; van Loon, 2007; Herranz et al., 2013). The idea is to provide a more complex motion patterns than constant rotation around one or two axes such that no directional preference remains. Ideally rotation should occur around all three spatial axes (*x*, *y*, and *z*, i.e., pitch, yaw, and roll) and would require a three gimbal or Cardan suspension. However, most RPM systems are based on two axes or an “altazimuth mount” such that the two axes are mounted perpendicular to each other (**Figure 3**). This arrangement is sufficient to position any object on the experimental platform in any desirable direction (i.e., the vector normal to the experimental platform can point in any direction). Thus, seedlings that develop on an RPM appear to grow randomly as achieved in spaceflight (**Figure 4**).

Randomness is achieved when the rotational angle differs between the two axes and changes over time. While these systems provide the best gravity compensation, they do so despite apparently exceeding the maximum permissible angular acceleration (approx. 30 deg s^−1^ for a 10-cm radius). Apparently better results are obtained when the sum of both axes movements exceeds 60–80 deg s^−1^ (Brungs et al., 2016). While this puzzling observation deserves future studies, it also highlights some postulated gravisensing mechanisms, namely that the movement of the suspected gravity sensors (starch-filled amyloplasts; Kiss, 2000) is sensitive to mechanostimulation, and thus describes dynamic gravisensing (Hasenstein, 2009).

**Figure 5:**
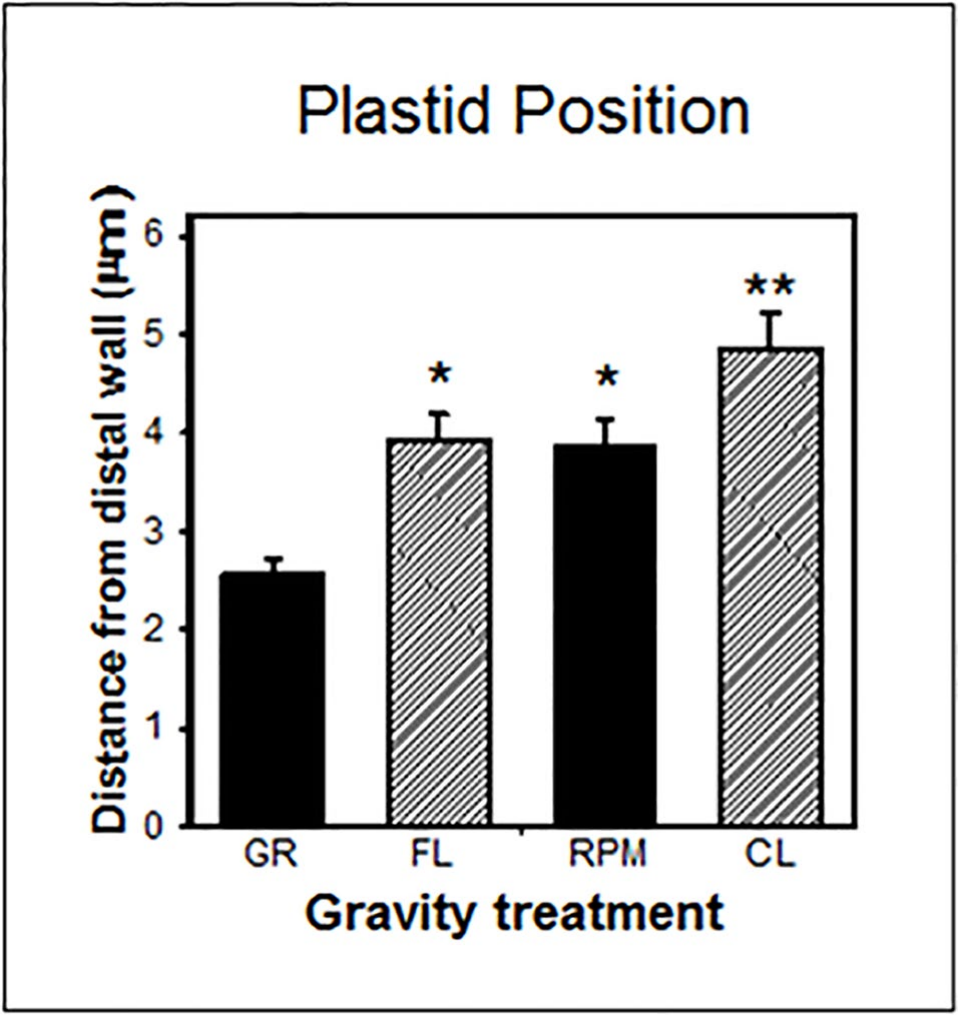
Plastid position in central columella cells of root tips of *Arabidopsis* seedlings grown on the ground (*GR*), during spaceflight (*FL*), on a random positioning machine (RPM), and on a clinostat (*CL*). These cells are involved in gravity perception (Kiss, 2000). There is no statistical difference (*P* > 0.05) between the FL and RPM samples as indicated by *, while the FL samples are significantly different (*P* < 0.05) from the CL specimens as indicaated by ** and determined by an ANOVA followed by a Tukey post-test.

Nevertheless, in gravity-perceiving root columella cells, the position of amyloplasts was similar in weightlessness in spaceflight and on the RPM, but was significantly different between spaceflight and two-axial clinostats (**Figure 5**). For *in vitro* systems like *Arabidopsis* cell cultures, it is important to realize that in fluid-filled experimental containers, there is also a fluid shear applied to the cells (Leguy et al., 2017). This issue can be mitigated by increasing the cell substrate viscosity (Kamal et al., 2019).

Thus, the RPM can be a useful proxy for weightlessness for certain biological parameters, as shown in studies with plant cells, *Drosophila*, and mammalian cell cultures (Kraft et al., 2000; Herranz et al., 2010; Wuest et al., 2015). In addition, due to the difficulty, availability, and cost of spaceflight experiments, the RPM may in fact be one of the best substitutes/analogs especially when this instrument can potentially generate results comparable to those in true microgravity. This scenario is true especially when the changes in direction are faster than the response time of the object (e.g., plant body) to gravity (Borst and van Loon, 2009).

## Magnetic Levitation

In contrast to the various clinostats that attempt to randomize the effect of gravity by changing the direction of its vector, magnetic forces counteract the gravity force by a magnetic force that results from a magnetic gradient and the diamagnetic susceptibility of the object which together generate a force that can be equal to gravity (Geim et al., 1999; Kamal et al., 2016). Interestingly, based on the orientation of the magnetic core, this gradient exists in two opposing directions such that in a vertically oriented magnetic field the top gradient balances gravity effects on biological, i.e., diamagnetic material at the point where *F*_mag_ = F_g_. The opposite pole of the magnetic gradient also generates a 1-*g* force equivalent and therefore provides a 2-*g* equivalent (1 *g* attributed to the magnetic gradient in addition to the original gravity). While the effect of magnetic gradients and diamagnetic properties of the levitated object (e.g., frogs, seeds, or seedlings) balances the effect of gravity (i.e., stably suspend biological objects is space), the very strong magnetic field (about 15 T) and gradient is likely to affect the movement of charged particles (ions) and therefore alters the physiological conditions which affect gene expression (Paul et al., 2006). In addition, the small space in a magnet bore, the requirement to cool magnets while maintaining “room temperature” for biological objects, and to provide light, contributes to the complexity of magnetic levitation. The required strong magnetic field and gradient (about 1,400 T^2^/m) also require specialized magnetic systems that are expensive to operate.

Additional research is needed to determine which systems best mimic reduced gravity conditions, especially for plants that occupy a large volume and are therefore affected by any gradient of rotational, inertial, or magnetic conditions. Despite the above-mentioned complications, the ability to produce partial or even excess gravity forces makes magnetic gradients an attractive alternative to clinostat-based research. As indicated earlier, the precise and narrow space that corresponds to the desired level makes studies on whole plants problematic because the compensation point averages all forces acting on the levitated object by susceptibility, density, and distance. Thus, the most valuable aspect of high-gradient magnetic fields is the ability to precisely move (levitate) cellular organelles, such as statoliths in roots (Kuznetsov and Hasenstein, 1996), hypocotyls (Kuznetsov and Hasenstein, 1997), rhizoids (Kuznetsov and Hasenstein, 2001), and seedlings (Hasenstein and Kuznetsov, 1999). In addition, magnetic levitation has been shown to be a useful ground-based proxy for microgravity in a number of other systems including osteoblast cells (Hammer et al., 2009), *Drosophila melanogaster* (Herranz et al., 2012), and bacteria (Dijkstra et al., 2010).

## Centrifuges

Although it sounds somewhat counterintuitive, we can also explore the effects of microgravity by the application of centrifuges. This reduced gravity paradigm (RGP) is based on the premise that adaptations seen going from a hypergravity level to a lower gravity level are similar to changes seen going from 1 *g* to microgravity (van Loon, 2016). Using such a paradigm, we are not focusing on the absolute acceleration values but rather on the responses generated due to the change between the two accelerations levels. The premise of such an experiment is that the plant sample has to be adapted and stable to a higher gravity level such as 2 *g*. Then, as the *g*-level is lowered to 1 *g*, the plant will respond to this reduced gravity level. It is hypothesized that the processes in such adaptations are of the same type as one would see going from 1 *g* into free fall, although the magnitude might be different. Thus, this reduced gravity paradigm is best used for stable and steady systems at a certain higher *g* level combined with measuring a relatively fast responding phenomenon when reducing the acceleration load.

## Reduced or Partial Gravity Studies

Numerous studies on plant growth and development have been performed in space (Wolverton and Kiss, 2009; Vandenbrink and Kiss, 2016). In contrast, we know little about plant physiology in reduced gravity environments, which are less than the normal 1 *g* that characterizes Earth-based studies. Reduced gravity can also be termed partial-*g* or fractional-*g*. The exploration of the Moon and Mars will be important in the future and will rely upon optimized plant cultivation because plants will be essential for life support systems (Kiss, 2014). Therefore, it is important to develop new knowledge about the biology of plants at the lunar and Martian *g*-levels, 0.17 *g* and 0.38 *g*, respectively. Studies on plants in partial gravity environments also can provide new information on basic biological questions such as what is the threshold of gravisensing in plants (e.g., Kiss et al., 1989; Perbal, 2009; Duemmer et al., 2015).

To establish partial gravity on-board sounding rockets or orbiting laboratories, a centrifuge is needed to produce the desired accelerations. Centrifuges can be used to generate any acceleration from near zero to 1 *g*. Especially 1-*g* experiments are valuable as in-flight controls, which provide context for the analyses of spaceflight experiments (Vandenbrink and Kiss, 2016). Fortunately, there are several facilities on the International Space Station (ISS) that are equipped with centrifuges, and the ISS can be used to study partial gravity effects on plant development.

### Plant Responses in Reduced or Partial Gravity in Spaceflight

A series of experiments have recently been performed on the ISS with *Arabidopsis thaliana* and have focused on 1) the interaction between gravitropism and phototropism in microgravity and fractional gravity (Kiss et al., 2012; Vandenbrink and Kiss, 2016; Vandenbrink et al., 2016) and 2) identification of the threshold for gravity perception in roots in the wild-type and starchless (*pgm-1*) mutants (Wolverton, in progress). These experiments utilized the European Modular Cultivation System (EMCS) which had onboard centrifuges allowing for gravitational ranges from microgravity to small fractions of a *g* up to 1 *g* (Kiss et al., 2014). The EMCS was decommissioned in 2017, but international space agencies have developed hardware such as Cell Biology Experiment Facility (CBEF) and Biolab support research at fractional *g* (Brinckmann, 2012).

**Figure 6:**
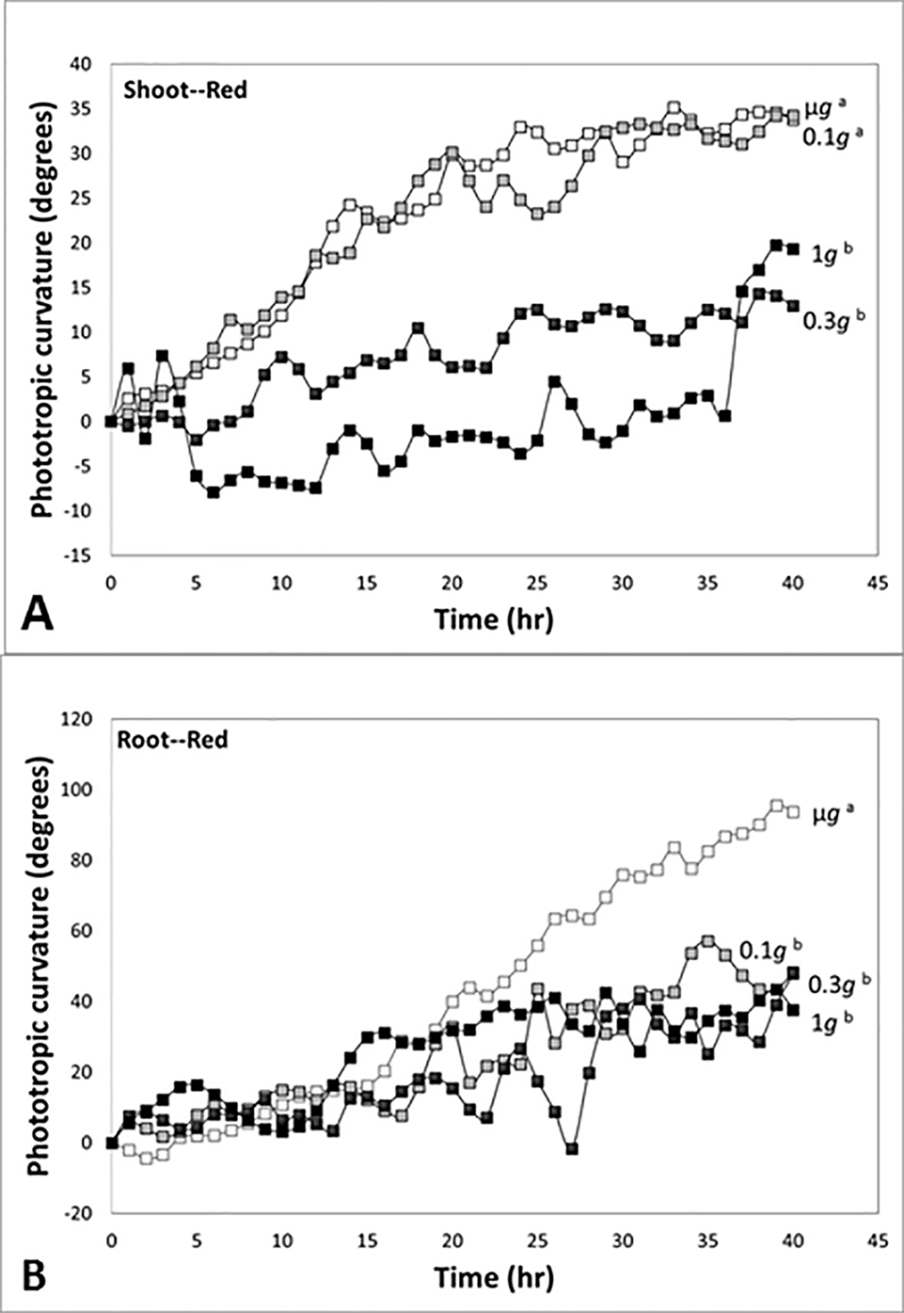
Time course studies of positive phototropic curvature in seedlings of *Arabidopsis* at indicated gravity levels in a spaceflight experiment. *Different letters* indicate significant differences among the plots. **(A)** Response of the shoot-like hypocotyls of *Arabidopsis* seedlings to red light. The response at 0.3 *g* was not significantly different from the value of the 1-*g* control, and there was attenuation of red-light phototropism at 0.3 *g* and 1 *g*. **(B)** Response of the roots of *Arabidopsis* seedlings to red light. The responses at 0.1 *g* and 0.3 *g* were not significantly different from the value of the 1-*g* control, and these values were attenuated compared to the robust response in microgravity. Figure is adapted from Kiss et al. (2012).

In experiments in which directional light and fractional gravity were applied simultaneously, Kiss and colleagues reported strong positive phototropism in response to unilateral red light in the stem-like hypocotyls and roots of plants grown in microgravity (Millar et al., 2010). In time course studies, shoots had positive phototropism in response to red light in microgravity and at 0.1 *g*, and the curvature was not significantly different between two gravity conditions (**Figure 6A**). However, the red-light-based phototropism at 0.3 *g* was not significantly different from the red-light phototropic response of the 1-*g* control, and there was significant reduction of red-light phototropism at 0.3 *g* and 1 *g* (see also Kiss et al., 2012).

In experiments with seedlings, roots exhibited a strong positive phototropism in response to unidirectional red illumination in microgravity conditions (**Figure 6B**). In contrast to the experiments with shoots, the red-light-based phototropic response in roots at 0.1 *g* was reduced and not significantly different from the red phototropic curvature at 0.3 *g* and 1 *g*. Thus, our fractional gravity experiments demonstrated a reduction of red-light-based phototropic curvature in the shoot-like hypocotyls at the level of 0.3 *g*, but the level of 0.1 *g* was enough to reduce the red-light-based phototropism in roots. This range of fractional gravity is approximately the same as the *g*-levels found on the Moon and Mars, 0.17 *g* and 0.38 *g*, respectively. Taken together, our results suggest that this range of reduced *g* represents a significant sensory threshold.

This hypothesis is being investigated further in a separate series of experiments designed to test the threshold force required to activate gravity sensing and response of *Arabidopsis* seedlings in the EMCS. Ground-based clinostat experiments have estimated the gravity perception threshold at or around 0.003 *g* (Shen-Miller et al., 1968; Laurinavicius et al., 1998; Duemmer et al., 2015). This threshold was tested in these space experiments, and the analysis currently is in progress. Extending these results to include the starchless mutant in addition to wild-type seedlings will allow for the comparison of gravity perception threshold in roots that lack sedimenting statoliths (Kiss et al., 1989), which we predict will require greater accelerations to activate perception and response in these seedlings.

### Plant Responses to Simulated Partial or Reduced Gravity Using Analogs

While the main focus of this paper has been on the simulation of microgravity, we also see that there is potential to use the analog devices to simulate partial or reduced gravity conditions that are found on the Moon and Mars (Kiss, 2014). This approach has been recently used in RPM studies of the effects of simulated partial gravity on the balance between cell growth and cell proliferation during early plant development (Manzano et al., 2018). In another recent study using *Arabidopsis* tissue culture cells, cell proliferation and growth were uncoupled under simulated reduced gravity also using an RPM (Kamal et al., 2018).

The results of these few studies are promising and encourage future exploration of simulated partial gravity for other biological systems. Successful application of partial gravity simulation could develop into new avenue of research. For example, the simulated Mars gravity of 0.38 *g* could be used in various biological studies to help prepare for a human mission to Mars.

## Conclusions and Future Directions

Numerous studies have compared the biological effects of clinostats and other microgravity analogs to space experiments (e.g., Brown et al., 1996; Kraft et al., 2000; Herranz et al., 2013; Huang et al., 2018). The experiments to date suggest that while these devices may be useful tools in some cases, there are great differences observed between plants that grow and develop on these devices and plants that are grown in weightlessness during spaceflight. In fact, rotation on certain types of clinostats may have deleterious effects in some biological systems (Hensel and Sievers, 1980; Kozeko et al., 2018; Ruden et al., 2018).

The conditions under which ground-based simulation can provide useful information and compare various gravitational regimens need to be systematically determined through carefully controlled experiments in which ground analog studies are compared with spaceflight experiments. A problem with past studies of microgravity simulators/analogs is that it can be difficult to compare results between spaceflight experiments to those of simulation devices (Herranz et al., 2013). Thus, ground-based experiments should be performed to maximize comparability between spaceflight and experimental ground-based devices. For example, in plant studies, factors to consider include identical seed stock, growth substrate, nutrient media, and light composition and intensity. In addition, containers should be identical for space and ground-based studies. This latter consideration can be made difficult by the reluctance of space agencies to provide access to their expensive spaceflight hardware (Kiss, 2015).

Nevertheless, in this era of the International Space Station, we must take advantage of its unique facilities to compare effects observed in clinostats and other space simulators. We also should use the centrifuges available on the ISS to systematically explore the effects of partial gravity on plant growth and development. Understanding plant biology in space under different gravity levels will be useful as we develop technologies needed for human habitation of other worlds.

## Author Contributions

JK wrote the first draft of the manuscript. CW, SW, KH, and JL also wrote and edited sections of the manuscript. All authors contributed to manuscript revision, read, and approved the submitted version.

## Funding

Financial support provided by grants from the National Aeronautics and Space Administration (NASA) to JK (#80NSSC17K0546 and #NNX12AO65G), CW (#NNX15AG55G), and KH (#80NSSC17K0344).

## Conflict of Interest

The authors declare that the research was conducted in the absence of any commercial or financial relationships that could be construed as a potential conflict of interest.
